# Dendritic Cell-Derived Exosomes may be a Tool for Cancer Immunotherapy by Converting Tumor Cells into Immunogenic Targets

**DOI:** 10.3389/fimmu.2014.00692

**Published:** 2015-01-19

**Authors:** Graziela Gorete Romagnoli, Bruna Barbosa Zelante, Patrícia Argenta Toniolo, Isabella Katz Migliori, José Alexandre M. Barbuto

**Affiliations:** ^1^Laboratory of Tumor Immunology, Department of Immunology, Institute of Biomedical Sciences, University of São Paulo, São Paulo, Brazil; ^2^Center for Cellular and Molecular Studies and Therapy (NETCEM), University of São Paulo, São Paulo, Brazil

**Keywords:** dendritic cells, exosomes, cancer immunotherapy, immunomodulation, tumor immune response, tumor cell immunogenicity

## Abstract

Dendritic cells (DCs) have been attracting attention in cancer immunotherapy because of their role in inducing and modulating effective immune responses. Besides the direct contact with other cell types and the secretion of cytokines, it is becoming clear that nanovesicles, such as exosomes (Exo), secreted by DCs also have a role in their function. Conversely, tumor-derived Exo carry antigens and have been used as a source of specific stimulus for the immune response against tumors. At the same time, several works have shown that different cells types incorporate DC-derived Exo (DC-Exo), resulting in modifications of their phenotype and function. Since DC-Exo carry many of the immune function-associated molecules of DCs, their incorporation by tumor cells could turn tumor cells into immunogenic targets. We have, therefore, treated human breast adenocarcinoma cells (SK-BR-3) with DCs-Exo and used these to stimulate previously SK-BR-3-primed CD3^+^ T-cells. Sensitized T-cells cultured with DC-Exo-treated tumor cells showed a significantly higher percentage of IFN-γ-secreting cells (as measured by ELISPOT), when compared to the frequency of cells responding to non-DC-Exo-treated cells. These data show that the incorporation of DC-Exo by the tumor cells increased their ability to activate T-cells for a possibly more effective response, thus showing that DC-Exo may become another tool in cancer immunotherapy.

## Introduction

Dendritic cells (DCs) are key players in the immune response; they are able to capture antigens with their pattern-recognition receptors, process and present them to *naïve* T-cells, inducing their activation (1, 2), thus, building an essential bridge between innate and adaptive responses. The central role that DCs play in the immune response, and the possibility of their *in vitro* generation has pathways for immunotherapy, in particular, for the treatment of cancer (3–8). However, the use of DCs outside clinical studies is hampered by the difficulties inherent to cell therapy strategies and, furthermore, in the case of DCs specifically against cancer, also by the compromised function of these cells in cancer patients (9–12). Not surprisingly, therefore, the general appraisal of DC-based strategies against cancer has been negative (10, 13). On the other hand, tumor cells do present potentially immunogenic antigens (14), which, when recognized by T-cells in immunotherapeutic approaches, seem to be associated with lasting tumor remissions (15). Therefore, strategies aimed at exposing tumor antigens to the immune system, bypassing the need for very active DCs, but in such a way that it leads to the establishment of T-cell responses, would be a potentially effective approach to harness the immune system to fight cancer.

In this context, therefore, it is relevant to note that, as most other cell types, DCs secrete nanovesicles, among which are the exosomes (Exo) (16–19). Exo are secreted vesicles that originate in the late endosomal compartment and result from the fusion of multivesicular bodies with the plasma membrane (20) and which can be acquired by other cells, at least in *ex vivo* cell cultures (21–24). These nanovesicles contain membrane proteins and genetic material, which, upon capture by other cells, contribute to the intercellular communication in the body (25–27). In fact, membrane traffic between DCs via Exo has been shown to occur (22), and Exo-carried antigens can be reprocessed for presentation or simply transferred directly to the membrane, in a process called cross-dressing (28). Furthermore, Exo transfer has been reported also to happen between cells of different types (25, 29, 30). Indeed, we demonstrated previously that Exo originated from DCs may be incorporated by tumor cells *in vitro* and that these tumor cells, after treatment with DC-derived Exo (DC-Exo), expressed molecules involved with antigen presentation, such as HLA-DR and CD86 (21).

Therefore, in this paper, we investigated if DC-Exo have the capacity to turn tumor cells into better targets for the immune system. We show that, indeed, DC-Exo treated tumor cells are able to induce tumor-sensitized T-cells to secrete higher levels of IFN-γ than non-DC-Exo-treated tumor cells. This observation supports our hypothesis and indicates that, as a minimum, DC-Exo used in cancer immunotherapy may act as a means to sensitize tumor cells to other immune effectors, thus enhancing the effectiveness of different immunotherapeutic approaches.

## Exosomes from Dendritic Cells and Their Role in Anti-Tumor Response

Raposo et al. (20) were the first to describe that Exo (originating from EBV-transformed B cells) contained functional MHC-II molecules, which carried peptides to which the cells were exposed and were able to stimulate peptide-specific CD4^+^ T-cells. From this initial observation, many others indicated a role for Exo in immune response to various stimuli, including tumors. Actually, in a mouse model, DC-Exo, containing class I major histocompatibility antigens (MHC-I) complexed with tumor-derived peptides were shown to induce a cytotoxic T lymphocyte (CTL) response, which inhibited tumor growth and rejected established tumors (16, 19, 22), probably due to the incorporation of the Exo by host DCs *in vivo* (31). Also, in a clinical trial, ascites-derived Exo administered with GM-CSF induced CEA-specific-T lymphocytes (17), confirming the potential of Exo to carry and deliver effectively tumor antigens, as observed in various other settings (16, 18).

## Exosomes from DCs Could Turn Tolerogenic Tumor Cells into Immunogenic Targets?

Though the generation of effective T lymphocytes responses is one of the main goals of cancer immunotherapy and the use of DC-Exo has been shown to be a means to achieve this goal, directly (19, 32, 33) or indirectly, by their incorporation by the recipients DCs (31, 33), the effectiveness of these immune effector cells depends on other factors. Actually, this is well illustrated by the fact that tumor-specific immune responses are frequently detected in patients who, nonetheless, have a progressive disease. This observation, essentially, has challenged for a long time, the potential of immunotherapy to control cancer. However, more recent experimental and clinical data have led to the realization that the immune system can, indeed, control and, probably, eliminate tumors, if “properly” engaged (34). In various clinical settings, this “proper” engagement of the immune system has been achieved, so far, either by the elimination of immune checkpoints, with monoclonal antibodies directed to immunoregulatory surface molecules (35), or by “arming” T lymphocytes with tumor-specific chimeric receptors, a strategy that, likewise, bypasses physiological controls of the immune response (36). However, these strategies depend, on one hand, on the existence of tumor cell targets already recognized by the immune effectors and, on the other hand, on the establishment of strong enough interactions between the immune effectors and the tumor targets.

**Figure 1:**
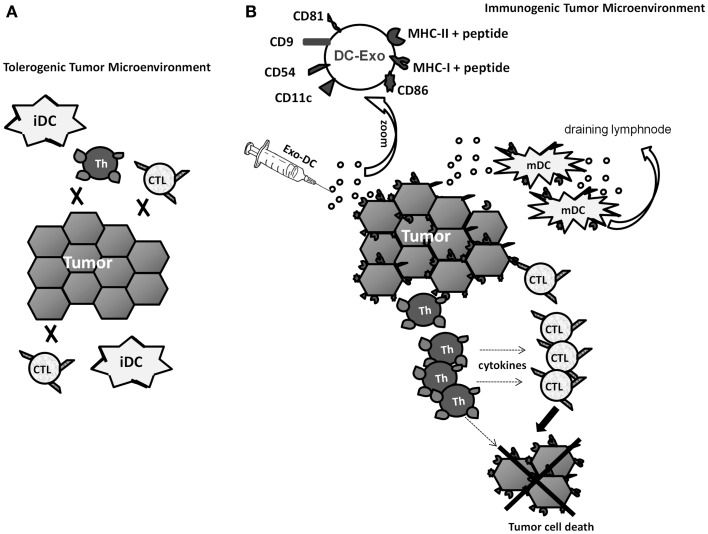
**Hypothetical scheme of the tumor microenvironment changed by treatment with Exo from immunogenic DCs (DC-Exo)**. **(A)** Low immunogenic tumor cells and immature DCs (iDCs) cannot induce specific-T lymphocyte response and, if such effectors do exist, they are not sufficiently stimulated by the tumor cells. **(B)** Enrichment of the tumor microenvironment with Exo-DC modifies tumor cells, enhances their immunogenic potential and turns them into better targets for any immune effector cell. Furthermore, immature DCs present in the tumor could also capture DC-Exo, acquire a mature phenotype and migrate to draining lymph nodes, where they could set up and amplify the immune response.

In this perspective, our observation that tumor cells incorporate DC-Exo and, thereafter, express various surface molecules involved in the interaction between antigen-presenting cells and T lymphocytes (21) gains a new relevance. Not only could DC-Exo-treated tumor cells gain antigen-presenting capabilities, which might induce *de novo* anti-tumor responses, but also such cells could become better targets to any immune effector cell, either naturally induced by the presence of the tumor in the patient or induced by immunotherapeutic approaches (Figure 1).

**Figure 2:**
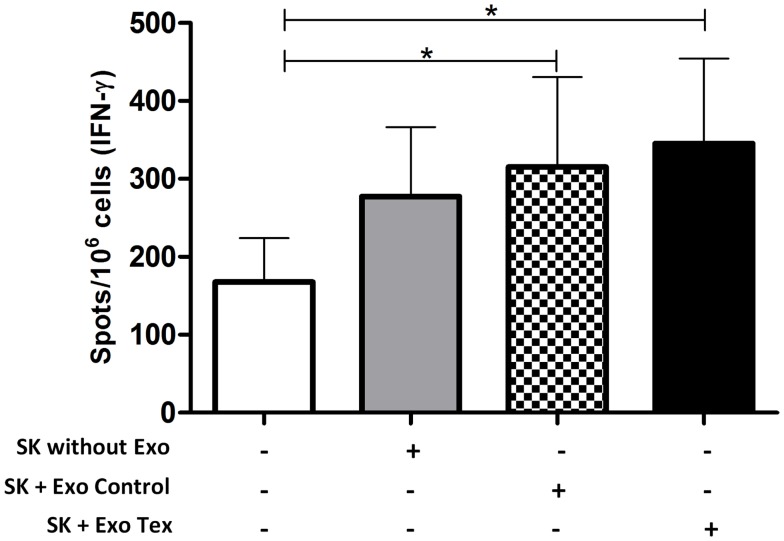
**IFN-γ-production by tumor-sensitized T-cells is enhanced when tumor target cells are treated with Exo obtained from DCs**. Cells of the human breast adenocarcinoma cell line SK-BR-3 were pre-treated or not with Exo (130 μg/10^6^ cells), obtained from control DCs (Exo Control) of from DCs exposed to tumor cell Exo (Exo Tex) and co-cultured for 2 days with previously sensitized CD3^+^ T-cells (autologous to the DC source of the Exo). After that, the cells were harvested and the number of IFN-γ-producing cells was quantified by ELISPOT. T-cells pre-sensitization was obtained by co-culture with autologous tumor cell exosome-treated DCs, for 14 days, in presence of IL-2 and IL-7. Average and SD of the number of cytokine-producing cells are represented (*n* = 6; ANOVA *p < 0.0028, flowing Dunnett’s Multiple Comparison Test).

To test this hypothesis, we sensitized human CD3^+^ T-cells against the breast carcinoma cell line SK-BR-3. Monocyte-derived DCs were pulsed with tumor cell Exo for 24 h, in the presence of IL-1, IL-6, TNF-α, and PGE_2_. Tumor-Exo were used as antigen source, since it was shown that they induce anti-tumor responses more efficiently than irradiated tumor cells, apoptotic bodies, or tumor cells lysate (37). Next, these DCs were co-cultured with autologous T-cells, in the presence of IL-2 and IL-7 for 14 days. After that, tumor-Exo sensitized T-cells were exposed to SK-BR-3 cells that were treated or not with DCs-Exo (Exo Control in the figure) or Exo obtained from DCs exposed to tumor Exo (Exo Tex in the figure). After 2 days, the IFN-γ-producing T-cells were quantified by ELISPOT (Figure 2). It is interesting to note that SK-BR-3 cells, alone, seemed to induce some cytokine production by sensitized T-cells, but this production was not statistically different from that of T-cells cultured alone. However, the treatment of the tumor cells with Exo (both Exo control and Exo Tex) increased this response to a significant level. It is noteworthy that these responses were modest, but nevertheless, significant, thus in agreement with our hypothesis: tumor cells treated with Exo derived from DCs became better targets for an already existing immune effector cell population – those T-cells that had been sensitized *in vitro* against the tumor cells, but whose response was not strong enough to raise above the background. Furthermore, when the Exo were obtained from DCs that were treated with tumor cell exosomes (Tex), the enhancement of IFN-γ production seemed to be even higher. Though speculative, it is possible to recognize, here, an antigenic enrichment of the targets, since cells may load their Exo not only with their co-stimulatory molecules but also with antigen-loaded MHC complexes (20), which would be captured by the tumor cells, hence turning these into even better targets for the antigen-specific-T-cells.

## Discussion

Tumor cells are poorly immunogenic and this has hampered the development of effective cancer immunotherapy. Yet, new insights on the role of different components of the immune response have broadened the field, so that, today, effective immune strategies to treat cancer are not longer seen as a remote possibility, but as a concrete breakthrough. However, though such approaches, as the use of checkpoint inhibitors or chimeric antigen receptor-transfected T-cells have reached astounding successes in some situations, the need for improvement is still pressing.

As depicted in Figure 1, even the presence (naturally or by transfer) of tumor-specific immune effectors within the tumor microenvironment may not be enough to hamper tumor growth and development. An immunosuppressive environment and targets that are poor in both antigens and co-stimulatory molecules may decrease significantly the effectiveness of tumor-specific responses. Therefore, any strategy that achieves the enhancement of tumor cell antigenicity or, even better, immunogenicity, should make it easier to control the disease in cancer patients. Such strategies could do so either by directly inducing tumor-specific responses, if the increase in tumor immunogenicity is strong enough, and, if not, by sensitizing tumors to borderline responses that, by themselves are not able to control the disease, but in face of more readily recognizable targets would do it.

Here, we tested the hypothesis that DC-Exo would perform such a role, increasing tumor cell antigenicity/immunogenicity. The transfer of information between cells via Exo is being recognized as a significant phenomenon for the body intercellular communication (38, 39), since Exo can interact with several cells types (26) and transfer information by means of fusion with the plasma membrane (40). Furthermore, this transfer seems not to be random, but controlled by molecules expressed by Exo and the “target cells,” as we have shown for the incorporation of DC-Exo by tumor cells, where the incorporation of DC-Exo by tumor cells was directly proportional to the expression CD9 molecules by the latter (21). In other setting, Exo capture, by the monocytes lineage, THP-1, has been shown to depend on toll like receptors, TLR-2 and TLR-4, leading to activation of NF-κB and STAT3, and to cytokine secretion (41).

Since Exo carry several molecules of their cells of origin (27), they would be a possible way to load tumor cells with antigen presentation-associated molecules from DCs. Indeed, we did observe the transfer of HLA-DR and CD86 molecules from DC-Exo to tumor cells (21). Furthermore, when we analyzed tumor cells treated with DC-Exo, we noticed that these had an increased expression of ICAM-molecules, which could facilitate their interaction with T lymphocytes, since the interaction between ICAM-I and LFA on lymphocytes accentuates TCR/MHC/peptide interaction (32).

Thus, we tested if SK-BR-3 tumor cells treated with DC-Exo would be able to induce a T-cell response more effectively. Accordingly, we observed that treatment of the tumor cell line with DC-Exo enhanced their ability to activate tumor-sensitized T-cells to secrete IFN-γ. We chose this cytokine because of its role in anti-tumor immune responses (42, 43) being associated with both CD8 and CD4 T-cell responses (44, 45), and essential for priming long-lived memory CD8^+^ T-cells (46, 47). It must be recognized, however, that the response we obtained though significant, when compared to the non-stimulated T-cell response, was modest, showing just a tendency to statistical significance, when compared to the response induced by the non-Exo-treated cells. This is, actually, in agreement with our hypothesis, since the expected action of the nanovesicles would be to enhance just enough the sensitivity of the targets to already existing immune effector mechanisms – and not, necessarily, the induction of new responses. Another issue that should be noted is that the clinical application of such a strategy would be restricted to situations where tumors might be directly accessed, since the systemic inoculation of Exo would hardly deliver them to the tumor cells, but, rather, to other cells along their distribution through the body.

On the positive side, however, it is worth noting that this strategy, of treating tumor cells with DC-Exo, eliminates the need of specific tumor antigens identification, since it transfers to the patients’ immune system this task. Through their association with DC-derived molecules, tumor antigens that would be poorly presented otherwise, would have their “visibility” increased, and, therefore, would be more likely to become effective targets of the patient’s immune response. Furthermore, the local injection of these DC-Exo could allow them to be captured also by the patient’s DCs in the tumor microenvironment. This would have a positive effect in the tumor immune response since in cancer patients, tumor-infiltrating DCs have functional deviations, maintaining an immature phenotype (9, 48–51). Thus, if these DCs incorporate Exo derived from immunogenic DCs, a phenomenon shown to occur (22), they might even become able to induce the response of tumor-specific *naïve* T lymphocytes.

Finally, even if the modifications of tumor cells immunogenicity caused by their incorporation of DC-Exo are not enough to give rise to *de novo* tumor-specific immune responses in the patients, they would, as we show here, turn the tumor cells into more effective targets to immune effectors induced by any other means. Thus, it is possible to suggest that treatment of tumors with DC-Exo could contribute to the effectiveness of any immunotherapeutic strategy, be it active, like vaccination protocols or passive, like the transfer of tumor-specific effectors. In face of this hypothesis and the data we obtained, we believe that this approach should receive consideration and be further investigated, in order to evaluate better its possible effectiveness, and to determine the most effective Exo doses, the length of its effect on tumor cells, and, mainly, the precise activation status of the DC, used as Exo source.

## Author Contributions

Graziela Gorete Romagnoli: project development, participation in all experiments, analyses, and discussions. Bruna Barbosa Zelante: participation in the experiments, discussions, and drafting the article. Patrícia Argenta Toniolo: participation in the experiments, discussions, and drafting the article. Isabella Katz Migliori: participation in the experiments and discussions. José Alexandre M. Barbuto: project development, discussions, drafting, and revising the article.

## Conflict of Interest Statement

The authors declare that the research was conducted in the absence of any commercial or financial relationships that could be construed as a potential conflict of interest.
